# Vaginal assisted NOTES hysterectomy in The Netherlands; A prospective cohort study

**DOI:** 10.1016/j.eurox.2024.100323

**Published:** 2024-07-02

**Authors:** Ilse P.W. Bekkers, Rebecca Henschen, Nicol A.C. Smeets, Huib A.A.M. van Vliet, Anne Damoiseaux, Martine M.L.H. Wassen

**Affiliations:** a Department of Obstetrics & Gynecology, Zuyderland Medical Centre Heerlen, Henri Dunantstraat 5, 6419 PC Heerlen, Limburg, the Netherlands; b Department of Obstetrics & Gynecology, Catharina Hospital Michelangelolaan 2, 5623 EJ Eindhoven, the Netherlands; c Department Obstetrics and Gynecology, Universitair Ziekenhuis Gent, Corneel Heymanslaan 10, 9000 Gent, Oost-Vlaanderen, Belgium; d GROW School for Oncology and Reproduction, Maastricht University Medical Centre, Maastricht, the Netherlands; e Maastricht University, Maastricht, the Netherlands

**Keywords:** Vaginal hysterectomy, VNOTES, Vaginal assisted NOTES hysterectomy, Laparoscopy

## Abstract

**Objectives:**

Vaginal assisted Natural Orifice Transluminal Endoscopic Surgery (NOTES) combines the benefits of vaginal and endoscopic surgery. This study presents the results of the first vaginal assisted NOTES hysterectomies (VANH) in The Netherlands.

**Study design:**

A prospective cohort study was performed in two non-academic teaching hospitals in The Netherlands. Data was collected from patients who underwent a VANH for benign indications between August 2019 and April 2023. Baseline characteristics and data of intra- and postoperative surgical outcomes were recorded and analysed. The VANHs were performed by four experienced vaginal and endoscopic gynaecological surgeons.

**Results:**

A total of 200 patients underwent a VANH. Indications were dysfunctional menstrual bleeding (61 %; n = 122), abnormal cervical cytology (15.5 %; n = 31), abdominal pain (11.5 %; n = 23), post ablation/sterilization pain syndrome (3.5 %; n = 7), uterine fibroids (5.0 %; n = 10), atypical endometrial hyperplasia (2.5 %; n = 5) and Lynch or BRCA gene mutation carriers (1.0 %, n = 2). The mean surgical time was 61.4 min ( ± 22.8 min) with a mean blood loss of 88 mL ( ± 89 mL) and a mean uterine weight of 150 g ( ± 112 g). In 2.0 % (n = 4) of the cases a conversion was necessary. Same day discharge (SDD) was feasible in 80.2 % (n = 105) of the patients planned in day-care. In 2.0 % (n = 4) an intra-operative complication and in 9.0 % (n = 18) a post-operative complication occurred.

**Conclusion:**

This study shows vNOTES to be a safe and feasible surgical technique and can be safely implemented with appropriate patient selection and skilled surgeons. It highlights the importance of surgeon awareness of the challenges inherent in the initial stages of the implementation of a new surgical technique when performing their first vNOTES procedures. Additional randomized clinical trials are needed to show superiority of vNOTES compared to traditional surgery.

## Highlights

• Vaginal Assisted NOTES hysterectomy is a safe and feasible surgical technique.
• Appropriate patient selection during a learning curve is important.
• More research to Vaginal Assisted NOTES hysterectomy is necessary.

## 1 Introduction

### 1.1 Background

Hysterectomy is the most performed gynaecological surgery worldwide [1], [2], [3], [4]. Vaginal hysterectomy (VH) is the preferred approach when feasible [3]. Since the introduction and implementation of the laparoscopic hysterectomy (LH), a decrease in performed VH was observed in The Netherlands (36 % in 2007 to 19 % in 2017) [5], [6].

As VH numbers decrease, there is a decline in skilled and experienced surgeons to perform a VH. A new endoscopic approach, ‘Natural Orifice Transluminal Endoscopic Surgery’ (NOTES) has gaining popularity. Vaginal NOTES (vNOTES) combines the benefits of laparoscopic and vaginal surgery, and has been increasingly used for a great variety of benign indications and broadens the indications of the VH [7], [8], [9].

The HALON trial was the first randomized controlled trial (RCT) comparing vNOTES hysterectomy to LH and demonstrated vNOTES to be non-inferior and allowed more women to be treated in day-care setting [10]. A systematic review and meta-analysis showed shorter operation time, hospital stay and less bloodloss with vNOTES compared to LH [11]. vNOTES might increase the trend towards vaginal surgery and is a technique to overcome the limitations of vaginal surgery. In addition to the regular benefits of vNOTES like no visible scars, less postoperative pain, less blood loss, decreased surgery time, no abdominal entry related risks, it enables more complex indications and allowing a complete exploration of the peritoneal cavity [10], [11]. For example, in patients with suspicion of mid and upper abdominal adhesions, vNOTES enables surgery without the need for adhesiolysis. Contained tissue extraction of adnexa or (enlarged) uteri is possible with vNOTES instead of conventional vaginal surgery [12]. Additionally, elective salpingectomy or adnexal surgery can be easily and safely combined with vNOTES hysterectomy, where it sometimes can be difficult with conventional VH [12]. Also in virgin women, vNOTES can be feasible if it is performed by an experienced vNOTES surgeon [13].

In The Netherlands, the Vaginal Assisted NOTES Hysterectomy (VANH) technique was first introduced and performed in August 2019 [14].

New surgical techniques are advised to be evaluated using the IDEAL framework [15]. This framework describes the stages of evaluation for a surgical innovation, respectively stage 0 the preclinical stage, stage 1 the idea, stage 2 development and exploration, stage 3 assessment (RCT), stage 4 evaluation involving long-term study and large-scale surveillance [15].

RCTs and systematic reviews are widely recognized as the gold standard methods for comparing clinical interventions [16], [17]. Several factors like surgeon training, team expertise, personal practice and the use of a variety of medical devices, pre- and postoperative management contributes to variation in the outcome of surgical procedures. Assessment of the long term monitoring according to IDEAL stage 4 provides an opportunity to obtain robust evidence about safety and effectiveness of this new technique.

This observational cohort study shows the data of the first performed VANH procedures of four experienced gynaecologists.

## 2 Methods

### 2.1 Study design, setting and participants

A prospective cohort study was performed in the Zuyderland Medical Centre and Catharina Hospital Eindhoven, two non-academic teaching hospitals. Data was collected from patients who underwent a VANH for benign indications between August 2019 and April 2023, starting from the first performed VANH. Patients were asked to participate in a RCT with approval number: NL76240.096.21 (METCZ20210035). If they did not want to participate in the randomization procedure, they gave informed consent to use the data for this cohort study. No study size was determined prior to the datacollection.

Eligible patients were 18 years or older, descensus uteri until at least halfway the vagina, no suspicion of pelvic adhesions or contra-indication for general anaesthesia. Exclusion criteria to undergo a VANH were any contra-indication for a VH, history of more than one caesarean section, rectal surgery, pelvic inflammatory disease, pelvic radiation, suspected (rectovaginal) endometriosis, and pregnancy. The VANH surgery was performed by four experienced vaginal and endoscopic trained gynaecologists who all performed the iNOTESS vNOTES course (MW, NS, HvV and AD), eventually also residents made surgical steps during the procedures.

#### 2.1.1 Surgical procedure

The VANH procedure consists of three phases as described earlier in the HALON trial protocol [10]. The first phase consists of an anterior and posterior colpotomy and transection and ligation of the uterosacral ligaments. The second phase, the endoscopic phase, is performed with a vNOTES port (GelPOINT V-Path; Applied Medical, Rancho Santa Margarita, CA). A opportunistic bilateral salphingectomy was performed in all cases. The third phase consists of closing the vaginal cuff, using a vicryl 1 running suture from both sacro uterine ligaments, as described in the HALON trial by Baekalandt [10].

#### 2.1.2 Treatment protocol

Patients received analgesia according to the local pain protocol with the preference of exclusion of opiods after surgery. Depending on the postoperative clinical condition, the surgery endtime, patient’s preference and patient’s medical history, women were discharged the same day after they had passed urine. In the Zuyderland Medical Centre the first five cases and in the Catharina Hospital Eindhoven the first ten patients were admitted for one day because of the introduction of a new technique.

### 2.2 Variables and data measurement

The following patient characteristics were collected pre-operatively: age, body mass index (BMI), medical history, vaginal parity, indication for hysterectomy.

Intra-operatively the following data were registered: conversion, duration of the total VANH surgery in minutes, duration of the three phases of the procedure in minutes, amount of blood loss in millilitres, weight of the uterus in grams and intra-operative complications.

Post-operative the following characteristics were collected: pain scores (Numeric Rating Scale (NRS)) one hour post-operative, at discharge of the patient and mean pain score during hospital stay, postoperative hospital stay in hours, procedures performed in same day discharge (SDD), complications in the first six weeks postoperative using the Clavien Dindo classification [18].

### 2.3 Statistical analysis

Statistical analysis was performed using SPSS Statistics version 25 (SPSS, NY, USA). The pre-, per- and postoperative data were collected and analysed with univariate descriptive analysis. The continuous variables were presented as mean (standard deviation (SD)), ordinal variables as mean (range) and nominal variables as frequencies (percentage).

## 3 Results

In the period of August 2019 until April 2023, a total of 200 VANH procedures for benign indications were performed. Table 1 presents an overview of the patient characteristics, compared to the data of the HALON trial.

**Table 1:** Patient characteristics.

	**Cohort (n = 200)**	**HALON trial vNOTES (n = 35)**
Age (years)	44.4 ( ± 8.9; 25-84)	46 (24-65)
BMI (kg/m2)	26.4 ( ± 5.2; 16.6-49.6)	27 (18-44)
**Previous abdominal surgery**	71 (35.5 %)	20 (57 %)
Endoscopic surgeries	61 (86.0 %)	not known
Laparotomic surgeries	5 (7.0 %)	not known
Endoscopic and laparotomic surgeries	5 (7.0 %)	not known
**Parity**		
Nulliparous	9 (4.5 %)	not known
Vaginal delivery	129 (64.5 %)	not known
Caesarean section	41 (20.5 %)	8 (23 %)
Vaginal and caesaran section	21 (10.5 %)	not known
**Indication for hysterectomy**		
Dysfunctional menstrual bleeding	122 (61 %)	5 (14 %)
Cervical dysplasia	31 (15.5 %)	4 (11 %)
Abdominal pain	23 (11.5 %)	-
Post ablation/sterilization pain syndrome	7 (3.5 %)	-
Uterine fibroids	10 (5.0 %)	17 (49 %)
Atypical endometrial hyperplasia	5 (2.5 %)	2 (6 %)
Lynch syndroom/BRCA gene mutation carrier	2 (1.0 %)	1 (3 %)
Adenomyosis	-	6 (17 %)

BMI = body mass index, BRCA = BReast CAncer

In 35.5 % (n = 71), patients had a medical history of abdominal surgery, excluding caesarean section (Table 1). Of these 71 patients, 86.0 % (n = 61) had only endoscopic surgery, 7.0 % (n = 5) had laparotomic surgery and 7.0 % (n = 5) had a history of endoscopic as well as laparotomic surgery.

Indications for a VANH were dysfunctional menstrual bleeding (61 %; n = 122), abnormal cervical cytology (15.5 %; n = 31), abdominal pain (11.5 %; n = 23), post ablation/sterilization pain syndrome (3.5 %; n = 7), uterine fibroids (5.0 %; n = 10), atypical endometrial hyperplasia (2.5 %; n = 5) and Lynch or BRCA gene mutation carriers (1.0 %, n = 2) (Table 1). Post-operative pathological examination outlines two cases (1 %) of endometrial cancer.

The mean total surgery time of a VANH was 61.4 min (SD 22.8 min). The mean duration of phase one was 17.7 min (SD 6.7 min), phase two 23.7 min (SD 11.8 min) and phase three 18.9 min (SD 11.4 min) (Table 2). The mean uterine weight was 150 g (SD 89 g, range 27–809 g) and the mean amount of blood loss was 88 mL (SD 112 mL) (Table 2). The mean NRS score during hospital stay was 2 (SD 2), 1 (SD 2) one hour postoperatively and 2 (SD 2) at discharge (Table 2).

**Table 2:** Surgical outcomes.

	**Cohort (n = 200)**	**HALON trial vNOTES (n = 35)**
Intra-operative outcomes		
Conversion rate, n (%)	4 (2.0 %)	0
Total duration of surgery (minutes)	61.4 ( ± 22.8)	41 ( ± 22)
*Phase 1*	17.7 ( ± 6.7)	not known
*Phase 2*	23.7 ( ± 11.8)	not known
*Phase 3*	18.9 ( ± 11.4)	not known
Uterine weight mean (grams)	150 ( ± 89)	206 (44-788)
Blood loss mean (mL)	88 ( ± 112)	not known
Intra-operative complications	4 (2 %)	1 (3 %)
Post-operative outcomes		
NRS score, mean (SD)		
*One hour postoperative*	1 (2)	not known
*At discharge*	2 (2)	not known
*Mean during admission*	2 (2)	not known
Complications 6 weeks post-surgery, n (%)	18 (9.0 %)	3 (9 %)
*Clavien Dindo grade 1*	6	1
*Clavien Dindo grade 2*	7	2
*Clavien Dindo grade 3a/b*	5	-
*Clavien Dindo grade 4*	-	-
*Clavien Dindo grade 5*	-	-
Re-admission, n	2 (1.0 %)	1 (3 %)

NRS = Numeric Rating Scale in pain. SD = standard deviation

Three VANH procedures (1.5 %) were converted to a LH and one (0.5 %) to an abdominal hysterectomy (AH), these cases are described in more detail (Table 3).

**Table 3:** Patient characteristics and reasons for conversions.

**Conversion number**	**Age (years)**	**BMI (kg/m2)**	**Indication**	**Previous pelvic surgery**	**Vaginal delivery**	**Number of surgery for surgeon**	**Reason conversion**	**Converted to**
1	52	38.5	Dysfunctional menstrual bleeding	No	Yes	3rd	No successful placement of Alexis wound retractor	LH
2	42	33.5	Abdominal pain	No	Yes	8th	Adhesions of sigmoid to pelvic wall	LH
3	46	24.6	Dysfunctional menstrual bleeding	Endometrial ablation, laparoscopic sterilization (filshie clips)	Yes	20th	Uterine fibroids and myoma in pouch of Douglas	LH
4	46	20.0	Dysfunctional menstrual bleeding	One caesarean section	No	51th	Per-operative bladder injury	AH

In four (2.0 %) procedures a cystotomy occurred intra-operatively.

The first case was a 41-year-old multipara with a history of one caesarean section and a BMI of 28.3 kg/m^2^. It was the 19th surgery for this surgeon. The cystotomy was vaginally sutured by the urologist.

The second case involved a 46-year-old multipara, with a history of one caesarean section and a BMI of 20 kg/m^2^. It was the 51th surgery for this surgeon. The surgery was converted to an AH and the cystotomy was sutured by the urologist.

The third case was a 49-year-old multipara with a BMI of 31.2 kg/m^2^ and a history of an endometrial ablation. It was the second surgical procedure for this surgeon. The urologist performed vaginal correction of the cystotomy.

The fourth case was 55-year-old multiparous patient with a BMI of 29.4 kg/m^2^, with a history of an endometrial ablation; this was the 19th procedure for the surgeon. The gynaecologist performed vaginal suturing and closure of the cystotomy.

A total of 80.2 % (n = 105) of the patients eligible for SDD were discharged the same day of surgery.

Before surgery, 69 patients (34.5 %) were scheduled to stay overnight for several reasons, while 131 (65.5 %) were eligible for SDD.

The remaining 19.8 % (n = 26) eligible for SDD but who stayed overnight, were admitted due the following reasons; postoperative nausea (n = 6), postoperative pain (n = 7), bladderretention (n = 3), thrombophlebitis (n = 1), intra-operative conversion or complication (n = 2), postoperative haemorrhage (n = 2), dizziness (n = 3), urticaria (n = 1), migraine post-operative (n = 1). Patients who stayed overnight had a mean hospital stay of 30.4 h (SD 18.3 h).

Postoperative complications as presented in Table 2 within the first six weeks after surgery were observed in 9.0 % (n = 18) of which 13 Clavien Dindo grade 1–2 complications (n = 7 urinary tract infection; n = 3 vaginal vault hematoma, n = 2 retention bladder, n = 1 thrombophlebitis) and five Clavien Dindo grade 3B complications (n = 4 postoperative haemorrhage; n = 1 vaginal vault dehiscence).

Three cases with postoperative haemorrhage presented one day postoperative, during their hospital stay. All three cases required a re-laparoscopy to resolve the haemorrhage and recovered without complaints. The fourth case presented with heavy vaginal bleeding of the vaginal cuff four weeks after the surgery. The bleeding required vaginal surgery. One patient had a vaginal vault dehiscence with herniation of the bowel 6 weeks post-operatively which was laparoscopic and vaginally repaired. Two patients were re-admitted due to complaints of pain without cause.

## 4 Discussion

The results of this prospective cohort showed the first data of VANH in The Netherlands, with a mean surgical procedure time to perform a VANH of 61.4 min, conversion rate of 2 %, an intra-operative complication rate of 2 % and post-operative complication rate of 9 %. Same day discharge was eligible in 80.2 % of the study group.

The study population was comparable regarding age, BMI and percentage of caesarean section in history with the HALON-trial [19]. Mean surgery time was longer (61 versus 41 min) and mean uterine weight lower (150 g (range 27–809) versus 206 (range 44–788)) in our study compared to the HALON trial [10]. However, when Baekelandt *et al.* initially conducted a feasibility study involving 10 patients, using a self-constructed single port device, mean operative time was 97 min with an average uterine weight of 132 g (range 51–353) [20]. The HALON trial was performed after the surgeon had completed 200 procedures.

The intra-operative complication rate of 2.0 % observed in our study is comparable with results in literature, which reported a complication rate of 3 % [10], [11], [19]. The post-operative complication rate in our study was 9.0 %, which is comparable with the HALON trial [10].

Baekelandt et al. recently published the complication rate of 1000 patients, which included vNOTES 73 % hysterectomy surgery. In the hysterectomy group the total complication rate was 4.9 % of which 1.3 % intra-operatively and 3.8 % post-operatively [19]. Regarding Clavien Dindo grade III complication rate this was comparable between the large cohort study (1.5 %) of Baekelandt and this cohort (2.5 %).

Small differences can be attributed to the fact that this cohort concerns the learning curve of four unexperienced vNOTES surgeons, whereas the study of Baekelandt et al. represents the data of a single very experienced vNOTES-surgeon and the learning curve of vNOTES was already fulfilled before recruiting data [19].

Our study demonstrated a total conversion rate of 2.0 %, which is lower than reported conversion rates in literature for VH and LH procedures [21], [22]. A conversion rate of 0.4 % was observed in the complication data of 1000 patients with strict patient selection, of which most conversions are to conventional laparoscopy (3/4) and one to laparotomy [19].

Three of four conversions in this cohort study occurred in the first 20 surgeries per surgeon. This can possibly contributed due to a poor patient selection. These cases represented one case of high BMI (BMI 38.5 kg/m^2^), one case with adhaesions of sigmoid to pelvic wall and the last had a myoma in the pouch of Douglas and uterine fibroids, before achieving enough surgical competence in a new surgical technique. A study assessing the learning curve of vNOTES in an experienced surgeon supposes surgical competence after 20 cases [23]. After 100 cases acquisition of competence and after 180 cases proficiency and acquisition was reached [23]. This reflects the initial surgical learning curve of the technique with a decrease of surgical time correlated to an increase in case load of the surgeon.

More than 80 % of the patients who were scheduled for SDD in this study were indeed discharged at the same day of the surgery, which is comparable with SDD rates of 77 % in literature [10], [24]. Moreover, the mean post-operative hospital stay for patients in this cohort was shorter than that reported for VH (1.13 to 2.2 days) [25], [26], [27], [28], [29], as well as LH [10]. A recent study comparing LH versus VANH for large uteri also showed a significant shorter hospital stay in favor of the VANH group [30].

The strength of this study, is that this is the first cohort study with a volume of 200 cases during the learning curve of four gynaecologists. This cohort study reflects the results of vNOTES in a general population and therefore represents good generalizability for surgeons who have the intention to start and implement vNOTES surgery.

However, some limitations should be mentioned. The study was performed during the COVID pandemic, which made it hard to include patients because elective surgery was less performed, but also a longer interval between surgeries per surgeon which may have had effect on the speed of the learning curve of the surgeons. Because this is a prospective cohort study, there is a higher risk for measurement and selection bias. In this cohort, all patients were operated by the same surgeons, with similar experience. It is well-known, that surgical load correlates with a better surgical outcome [31].

## 5 Conclusion

This study shows VANH to be a safe and feasible surgical technique at implementation in two non-experienced vNOTES centres with appropriate patient selection and skilled surgeons. It highlights the importance of surgeon awareness of the challenges inherent in the initial stages of the implementation of a new surgical technique when performing their first vNOTES procedures. It is important to carefully select appropriate patients based on uterine size, previous medical history, and BMI. Experienced vNOTES surgeons, can broaden the indications for vNOTES. Further research is required to delineate the indications for vNOTES and to compare its efficacy to other conventional surgical techniques such as laparoscopic hysterectomy and vaginal hysterectomy. The ongoing RCT in the Netherlands comparing vNOTES with vaginal hysterectomy will provide valuable insight into this issue.

## Funding

This study has no funding.

## CRediT authorship contribution statement

**Martine Wassen:** Writing – review & editing, Writing – original draft, Supervision. **Rebecca Henschen:** Writing – review & editing, Writing – original draft, Methodology. **Nicol Smeets:** Writing – review & editing. **Huib van Vliet:** Writing – review & editing, Supervision. **Anne Damoiseaux:** Writing – review & editing. **Ilse Bekkers:** Writing – original draft, Validation, Resources, Project administration, Methodology, Investigation, Formal analysis, Data curation, Conceptualization.

## Declaration of Competing Interest

Ilse Bekkers: none Rebecca Henschen: none Nicol Smeets: Received fees from Applied Medical on an hourly basis for lectures on the vNOTES technique. All the fees were donated to a foundation which promotes research in obstetrics and gynecology. Huib van Vliet: Received fees from Applied Medical on an hourly basis for lectures on the vNOTES technique. All the fees were donated to a foundation which promotes research in obstetrics and gynecology. Anne Damoiseaux: Received fees from Applied Medical on an hourly basis for lectures on the vNOTES technique. All the fees were donated to a foundation which promotes research in obstetrics and gynecology. Martine Wassen: Received fees from Applied Medical on an hourly basis for lectures on the vNOTES technique. All the fees were donated to a foundation which promotes research in obstetrics and gynecology. Member of the vNOTES faculty Europe.
